# Clinical characteristics and cytokine profiles for early prediction of severe *Mycoplasma pneumoniae* pneumonia in children: a prospective cohort study

**DOI:** 10.3389/fped.2026.1862535

**Published:** 2026-07-10

**Authors:** Jiayi Xue, Tao Ai, Yinghong Fan, Cheng Xie, Wanmin Xia, Li Wang

**Affiliations:** Department of Pediatric Respiratory Medicine, Chengdu Women’s and Children’s Central Hospital, School of Medicine, University of Electronic Science and Technology of China, Chengdu, China

**Keywords:** child, cytokines, *Mycoplasma pneumoniae*, nomogram, pneumonia, prediction model

## Abstract

**Introduction:**

To investigate the clinical characteristics and cytokine profiles of children with *Mycoplasma pneumoniae* pneumonia (MPP) and to establish a predictive nomogram for the early recognition of severe *Mycoplasma pneumoniae* pneumonia (SMPP).

**Methods:**

This prospective study enrolled 445 children with MPP admitted to the Department of Pediatric Respiratory Medicine of Chengdu Women's and Children's Central Hospital between December 2022 and December 2023. Based on disease severity, patients were assigned to the mild MPP (MMPP, *n* = 190) and SMPP (*n* = 255) groups. Clinical features and laboratory parameters were compared between the two groups.Binary Logistic regression analysis was used to identify the independent risk factors of SMPP, and a predictive nomogram was constructed based on these factors. The discrimination of the model was evaluated by the area under the receiver operating characteristic curve (AUC) and calibration curve. In addition, 42 children were randomly selected from each group using stratified random sampling, and serum cytokine levels were measured by enzyme-linked immunosorbent assay.

**Results:**

Among the 445 children, 255 cases (57.3%) developed SMPP. Multivariate regression analysis showed that wheezing (OR=4.016, 95% CI: 1.609–10.029), shortness of breath (OR=4.717, 95% CI: 1.290–16.008), D-dimer (OR=3.032, 95% CI: 1.926–4.773), CRP (OR=1.033, 95% CI: 1.018–1.048), fever (OR=3.432, 95% CI: 1.658–7.105), and age (OR=1.114, 95% CI: 1.015–1.223) were independent risk factors for SMPP (all *P* < 0.05). The constructed nomogram prediction model had an AUC of 0.818 and the calibration curve indicated good predictive ability. In the cytokine sub-cohort (*n* = 84), serum levels of HMGB-1, TFEB, MCP-1, MCP-2, MCP-3, MCP-4, and TNF-α were significantly higher in the SMPP group than in the MMPP group (all *P* < 0.05).

**Conclusion:**

A predictive nomogram incorporating wheezing, shortness of breath, fever, D-dimer, CRP, and age was established for early risk stratification of SMPP in hospitalized children. Multiple serum cytokines were significantly elevated in SMPP patients, and their potential clinical utility warrants further investigation in larger cohorts.

## 1 Introduction

*Mycoplasma pneumoniae* (MP) is a key player in the realm of pathogens responsible for both upper and lower respiratory tract infections, occupying a prominent place in the spectrum of bacteria that cause community-acquired pneumonia (CAP). Epidemiological data indicate that approximately 40% of CAP cases in children aged 5 and under are attributable to MP infection (1). The typical signs of *Mycoplasma pneumoniae* pneumonia (MPP) include fever,cough, and may also be accompanied by expectoration, wheezing, chills, headache, etc, and may be complicated with pulmonary and extrapulmonary complications. In severe cases, acute respiratory distress syndrome may develop, which can be life-threatening (2). However, it is difficult to identify children who may develop severe Mycoplasma pneumoniae pneumonia (SMPP) from symptoms during the early stage of the disease. Excessive immune responses during MP infection, including the excessive release of pro-inflammatory factors and activation of immune cells, are markers of the pathogenesis and disease progression of MPP. Dynamic monitoring of cytokines is therefore of great significance for understanding disease progression (3). The study period coincided with a critical phase in which the epidemiologic pattern of *Mycoplasma pneumoniae* (MP) infection in China changed markedly after the COVID-19 pandemic. In the second half of 2023, China experienced its first major MP outbreak following the relaxation of pandemic control measures, with severe cases reported frequently across regions. China published its first pediatric MPP diagnosis and treatment guideline in 2023, which was updated in 2025, both emphasizing the need to shift the focus toward earlier identification of severe disease (4). This study aims to explore early warning indicators for SMPP in children by comprehensively analyzing multiple aspects of data, including clinical manifestations, relevant laboratory indicators, and cytokine levels (HMGB-1, TNF-α, IL-6, IL-18, TFEB, MCP-1, -2, -3, -4) of children with SMPP. The aim is to equip healthcare professionals with more precise and reliable diagnostic suggestions, allowing for timely identification and immediate intervention of SMPP, improving treatment efficacy and elevating quality of life among pediatric patients.

## 2 Materials and methods

### 2.1 Patients

This study selected 445 children diagnosed with MPP in the Department of Pediatric Respiratory Medicine of Chengdu Women and Children's Central Hospital from December 2022 to December 2023, all of whom fulfilled established diagnostic criteria for MPP. In accordance with the severity of the disease, they were divided into 190 cases in the mild *mycoplasma pneumoniae* pneumonia (MMPP) group and 255 cases in the SMPP group.Using stratified random sampling method, with outcome groups (SMPP, MGMPP) and age groups (<3 years old, 3–5 years old, >5 years old) as stratification factors, the number of participants was allocated according to the proportion of each stratum in the total cohort. 42 cases were randomly selected from each group (a total of 84 cases) to form the cytokine detection sub-cohort.

### 2.2 Inclusion and exclusion criteria

#### 2.2.1 Inclusion criteria

According to guidelines outlined in the 2015 edition of the “Expert Consensus on the Diagnosis and Treatment of *Mycoplasma Pneumoniae* Pneumonia in Children “ and the 2019 “Diagnosis and Treatment Guidelines for Community-Acquired Pneumonia in Children (5, 6), a child is considered to have MPP if they display clinical manifestations or radiological evidence in keeping with pneumonia and satisfy a minimum of one of the subsequent conditions:(1) Serological test: a single serum MP antibody titer ≥ 1:160 (using passive particle agglutination method), or a fourfold or greater increase in MP antibody titer in two serum samples; (2) Pathogen test: positive detection of MP-DNA/RNA in respiratory specimens (including nasopharyngeal swabs, bronchoalveolar lavage fluid, etc.).

#### 2.2.2 Exclusion criteria

(1) Coexisting chronic respiratory inflammatory diseases, primary/acquired immune deficiency, immunosuppression, or chronic underlying diseases of other organ systems;
(2) Having received immunomodulatory or immunosuppressive therapy within past 3–6 months;
(3) A history of severe underlying health issues or significant organ dysfunction;
(4) Incomplete clinical data.

#### 2.2.3 Grouping criteria

(1) MMPP group: Meeting the above-mentioned MPP diagnosis criteria but not reaching the severe condition standard;
(2) SMPP group: On the basis of MPP diagnosis, presenting any of the following manifestations: ① Poor general condition (such as drowsiness, irritability, and other consciousness changes); ② Presence of dehydration signs or eating disorders; ③ Appearance of breathing difficulty symptoms (such as moaning, flaring of the nostrils, trilateral depression, and other breathing difficulty signs); ④ Resting state finger pulse oxygen saturation ≤ 92%; ⑤ Chest imaging shows unilateral lung lobe involvement ≥ 2/3, multiple lung lobe infiltration or moderate to large pleural effusion; ⑥ Complications of extrapulmonary system damage (such as cardiovascular, neurological, hematological, and other system complications).

### 2.3 Research methods

#### 2.3.1 Collection of clinical manifestations and laboratory tests

(1) Basic information: age, gender, weight;
(2) Clinical manifestations: fever, peak temperature, fever duration, cough, wheezing, shortness of breath;
(3) Laboratory test indicators: peripheral venous blood was collected within 24 h of admission to test procalcitonin (PCT), white blood cell count (WBC), D-dimer (D-D), neutrophil (N) percentage, C-reactive protein (CRP); Pathogen detection: Gel particle agglutination method was used to detect the MP antibody titer in serum, sputum culture, bronchoalveolar lavage fluid culture to detect pathogens, and nucleic acid detection of respiratory pathogens (including respiratory syncytial virus, rhinovirus, adenovirus, MP, influenza A,B virus).

#### 2.3.2 Cytokine assay

In our previous study, through the analysis of the whole blood transcriptome of children with SMPP and MMPP, we selected cytokines with significant differences in expression levels. In this study, we measured the cytokines with significant differences. According to the instructions of the enzyme-linked immunosorbent assay (ELISA) kits provided by Shanghai Zhuocai Biotechnology Co., Ltd., we measured the levels of serumtumor necrosis factor (TNF)-*α*， high mobility group box 1 (HMGB-1), monocyte chemoattractant protein (MCP)-1, -2, -3, -4, transcription factor (TF) EB, and IL-6, -18.

### 2.4 Statistical analysis

The data evaluation was conducted using SPSS 27.0 and R 4.5.2. For categorical variables, descriptive statistics were performed using frequencies and proportions, and the differences between groups were analyzed using the chi-square test; for continuous variables that followed a normal distribution, the mean and standard deviation were selected as descriptive statistics indicators, and the independent sample t-test was used to evaluate the differences between groups; for continuous variables that deviated from a normal distribution, the median and interquartile range were reported, and the Mann–Whitney U test was used to compare the differences between groups. Variables with *P* < 0.05 in the univariate analysis were included in the multivariate binary Logistic regression (backward LR method) to screen independent risk factors and establish a nomogram. The discrimination of the model was evaluated by the area under the receiver operating characteristic curve (AUC). The calibration was intuitively assessed using the calibration curve graph. A *P* value > 0.05 was considered to be without statistical significance.

## 3 Results

### 3.1 Clinical manifestations and laboratory examination of children with SMPP and MMPP

#### 3.1.1 Comparison of general information

This study encompassed 445 children diagnosed with MPP. All children with MPP were treated and subsequently discharged upon recovery, with no fatalities reported. Based on the diagnostic criteria for SMPP,the patients were systematically classified into MMPP group and SMPP group. The MMPP group comprised 190 cases (83 males and 107 females), with an mean age of 5.88 ± 3.23 years. The SMPP group had 255 patients (118 males and 137 females), with a mean age of 6.64 ± 2.62 years. The mean age of children in the SMPP group was notably greater than that in the MMPP group (*P* < 0.01). Among children with SMPP, school-age children exhibited the highest incidence, with a statistically significant difference compared to the MMPP group (*χ*^2^ = 15.813, *P* < 0.01). There were no notable differences in gender distribution or weight between the two groups (*P* > 0.05) (Table 1).

**Table 1:** Baseline characteristics of the two study groups at the time of enrollment.

Parameter	SMPP(*n* = 255)	MMPP(*n* = 190)	Statistic (T/*χ*2)	*P* value
Age($\bar{x}$±s，year)	6.64 ± 2.62	5.88 ± 3.23	−2.637	0.009
Stage of age[n(%)]			15.813	<0.001
Infancy and toddler stage	24 (9.4)	43 (22.6)		
Preschool stage	60 (23.5)	45 (23.7)		
School stage	171 (67.1)	102 (53.7)		
Gender			0.295	0.587
Male	118（46.3）	83（43.7）		
Female	137（53.7）	107（56.3）		
Weight ($\bar{x}$±s，kg)	22.93 ± 8.93	22.48 ± 11.92	−0.45	0.653

Data are presented as the mean ± standard deviation, or the number (percentage). SMPP, severe *mycoplasma pneumoniae* pneumonia; MMPP, mild *mycoplasma pneumoniae* pneumonia.

#### 3.1.2 Clinical feature analysis

In this research, a notably higher percentage of children in the SMPP group presented with fever when contrasted with those in the other group, with this disparity reaching statistical significance. Within the SMPP cohort, high fever was prevalent, whereas moderate and low fevers occurred less often. Conversely, in the MMPP group, moderate fever was the predominant type, though high fever was also seen with some frequency. A statistically substantial difference in fever severity was evident when comparing the two sets of subjects. Additionally, variations were discerned across the SMPP and MMPP groups regarding the duration of fever, peak temperature, as well as the presence of wheezing and shortness of breath. However, no significant differences emerged among the two cohorts with regard to sputum production and mixed infection rates among the children. Furthermore, the magnitudes of neutrophils, CRP, D-D, and PCT were notably elevated in the SMPP group differing from the MMPP group（P＜0.05). WBC levels didn't differ substantially when comparing the two sets of subjects (*P* > 0.05) (Table 2).

**Table 2:** Clinical feature analysis of the two study groups.

Parameter	SMPP(*n* = 255)	MMPP(*n* = 190)	Statistic (T/χ2)	*P* value
fever [*n*(%)]	232 (91.0)	142 (74.7)	21.432	<0.001
Degree of fever [*n*(%)]			49.927	<0.001
<38.0℃	5 (2.2)	16 (11.3)		
38.1–39.0℃	62 (26.7)	76 (53.5)		
39.1–42℃	165 (71.1)	50 (35.2)		
fever duration [M(P25–P75)，day]	6 (5,7.88)	3 (2,4)	−11.63	<0.001
peak temperature ℃	39.467 ± 0.602	38.905 ± 0.63	−8.502	<0.001
Expectoration [*n*(%)]	225 (88.2)	173 (91.1)	0.915	0.339
Wheezing [*n*(%)]	41 (16.1)	17 (8.9)	4.885	0.027
Shortness of breath [*n*(%)]	37 (14.5)	4 (2.1)	20.029	<0.001
Mixed infection [*n*(%)]	46 (18)	30 (15.8)	0.389	0.533
WBC [M(P25–P75), ×109/L]	7.95 (6.2,10.16)	8.105 (6.122,10.6)	−0.261	0.794
N [M(P25–P75), %]	64.4（57.4，71.5）	59.55（48.53，67.65）	−4.564	<0.001
CRP [M(P25–P75), mg/L]	19.2 (7.2,38.3)	7.15 (3,19.12)	−6.59	<0.001
D-D [M(P25–P75), ug/ml]	0.7 (0.38,1.56)	0.38 (0.22,0.545)	−8.091	<0.001
PCT [M(P25–P75), ng/ml]	0.142 (0.087,0.266)	0.1 (0.05,0.163)	−4.924	<0.001

Data are presented as the mean ± standard deviation, or the number (percentage),ormedian (25th percentile, 75th percentile).SMPP, severe *mycoplasma pneumoniae* pneumonia; MMPP, mild *mycoplasma pneumoniae* pneumonia; PCT, procalcitonin; WBC, white blood cell; D-D, D-dimer; N, neutrophils; CRP, C-reactive protein; N, neutrophilic granulocyte percentage.

#### 3.1.3 Multivariate logistic regression analysis

Taking the occurrence of SMPP as the dependent variable and the single factors with significant significance as the independent variables, the study analyzed the independent influencing factors of SMPP occurrence. The results showed that wheezing [OR=4.016 (95% CI: 1.609, 10.029)], shortness of breath [OR=4.717 (95% CI: 1.29, 16.008)], D-D [OR=3.032 (95% CI: 1.926, 4.773)], CRP [OR=1.033 (95% CI: 1.018, 1.048)], fever [OR=3.432 (95% CI: 1.658, 7.105)], and age [OR=1.114 (95% CI: 1.015, 1.223)] were independent risk factors for SMPP occurrence (*P* < 0.05) (Table 3).

**Table 3:** Multivariate binary logistic regression analysis.

Variable	*β*	Standard Error	Wald	P	OR	95% CI	
						Lower	Upper
Wheezing	1.39	0.467	8.872	0.003	4.016	1.609	10.029
Shortness of breath	1.551	0.623	6.192	0.013	4.717	1.29	16.008
N	0.017	0.009	3.156	0.076	1.017	0.998	1.036
D-D	1.109	0.232	22.942	<0.001	3.032	1.926	4.773
CRP	0.032	0.007	18.289	<0.001	1.033	1.018	1.048
Fever	1.233	0.371	11.031	0.001	3.432	1.658	7.105
Year	0.108	0.047	5.201	0.023	1.114	1.015	1.223
Constant	−4.104	0.67	37.557	<0.001	0.017		

D-D, D-dimer; N, neutrophils; CRP, C-reactive protein; N, neutrophilic granulocyte percentage.

#### 3.1.4 Development of a predictive nomogram for SMPP

**Figure 1:**
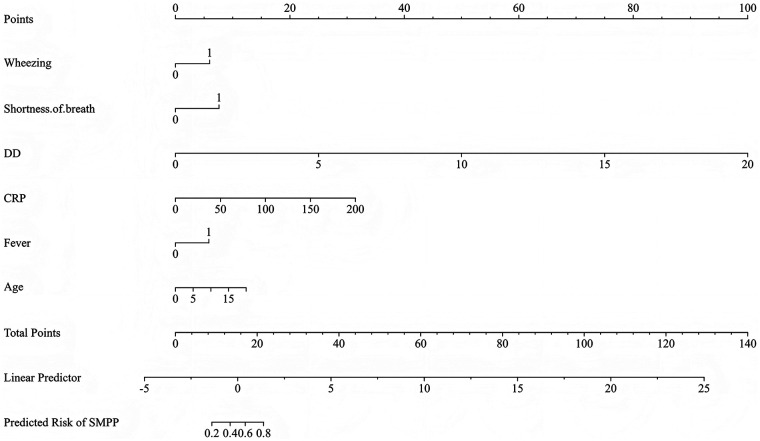
Predictive nomogram for SMPP.

**Figure 2:**
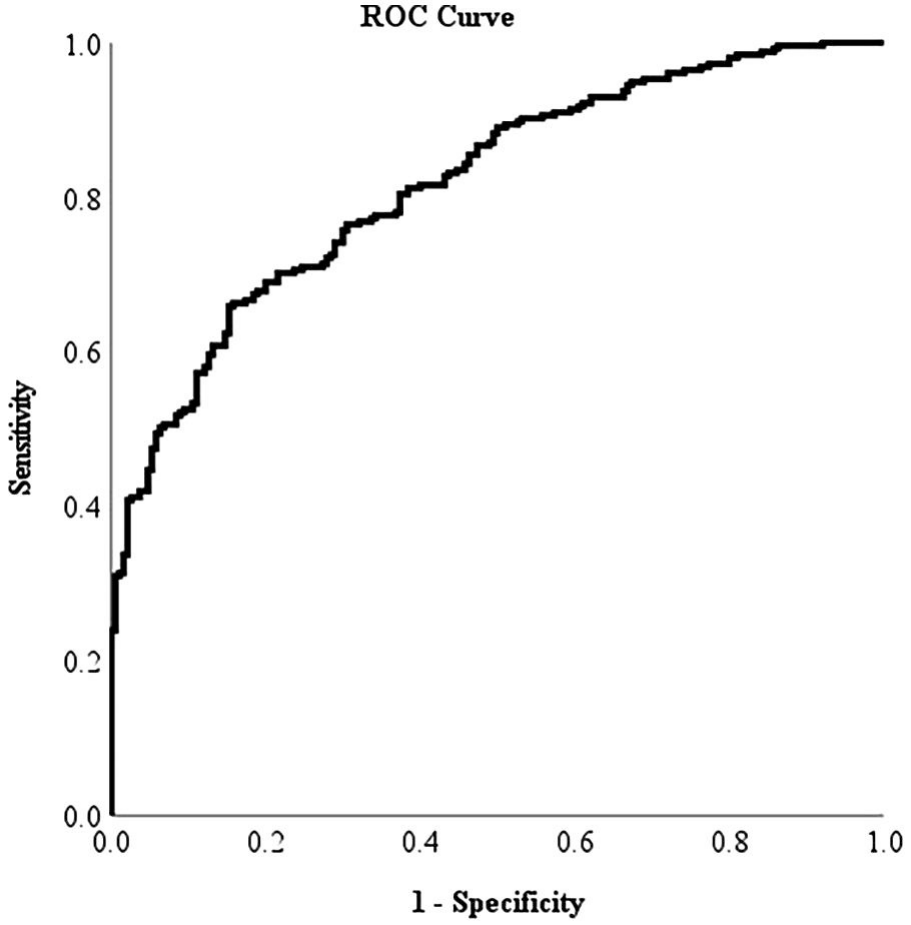
Receiver operating characteristic (ROC) curve of the combined prediction model.

**Figure 3:**
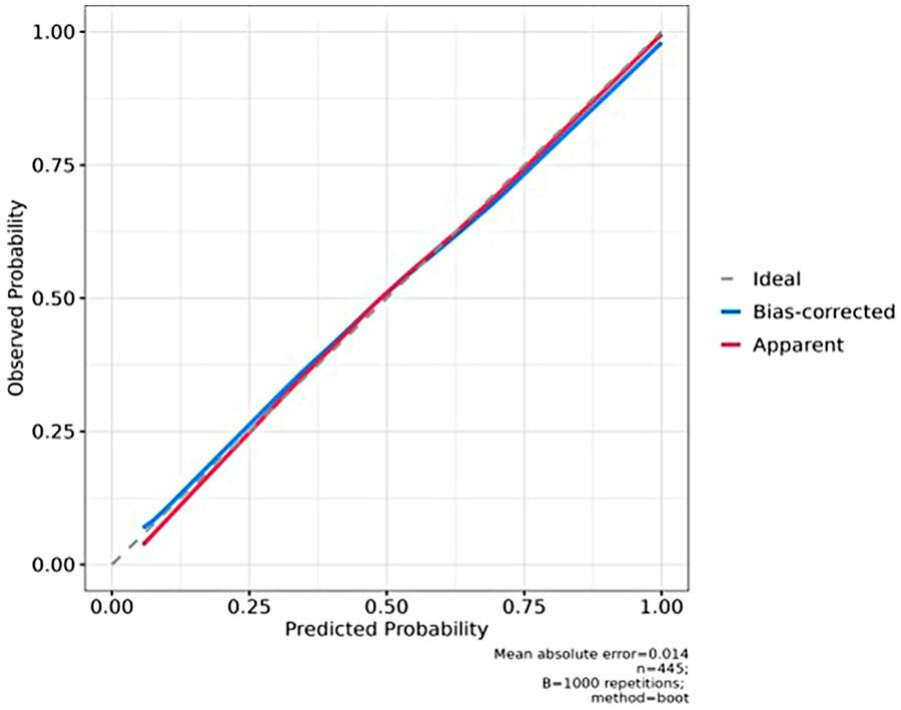
Calibration curve of the predictive nomogram.

Independent risk factors identified by multivariable logistic regression were integrated to construct a nomogram for predicting the probability of SMPP. The total points, calculated as the sum of the individual scores assigned to each predictor, correspond to the predicted risk of SMPP occurrence(Figure 1). The established nomogram prediction model had an area under the ROC curve of 0.818 (95% CI 0.78–0.856), and the calibration plot of the model further confirmed that there was a good consistency between the predicted probability and the actual observed probability (Figures 2 and 3).

### 3.2 Analysis of serum cytokine levels results

To validate the effectiveness of the stratified random sampling procedure, baseline characteristics were compared between the cytokine sub-cohort (*n* = 84) and the complete cohort (*n* = 445) (Supplementary Table 1), no statistically significant differences were observed between the two groups in terms of age, sex distribution, fever duration, length of hospital stay, proportions of wheezing and tachypnea, D-dimer, CRP, neutrophil count, PCT, and LDH levels (all *P* > 0.05). These findings confirm that the cytokine sub-cohort was demographically, clinically, and biochemically representative of the complete cohort, demonstrating the successful implementation of the stratified random sampling design. The proportions of HMGB-1, MCP-1, -2, -3, -4, TFEB, IL-6, IL-18, and TNF-α in the SMPP group exhibited a markedly greater magnitude compared to the MPP group (Table 4).

**Table 4:** Cytokine levels.

Parameter	SMPP (*n* = 42)	MMPP (*n* = 42)	Statistic (Z)	*P* value
HMGB-1[M(P25–P75),pg/mL]	1345.886 (1114.062,2158.390)	1018.465 (824.478,1331.075)	−4.088	<0.001
MCP-1[M(P25–P75),pg/mL]	23.507 (17.221,36.384)	16.075 (13.585,21.405)	−3.543	<0.001
MCP-2[M(P25–P75),ng/mL]	1.255 (0.8475,1.915)	1.02 (0.7975,1.365)	−2.255	0.024
MCP-3[M(P25–P75),pg/mL]	21.702 (16.567,28.862)	16.384 (14.687,20.885)	−3.095	0.002
MCP-4[M(P25–P75),pg/mL]	30.887 (22.325,40.219)	18.215 (14.426,24.305)	−4.12	<0.001
TFEB[M(P25–P75),pg/mL]	96.881 (69.158,139.498)	67.19 (54.72,85.029)	−3.315	0.001
IL-6[M(P25–P75),pg/mL]	7.416 (5.696,14.867)	6.842 (3.758,9.031)	−2.174	0.03
IL-18[M(P25–P75),pg/mL]	43.661 (31.347,84.667)	36.938 (23.098,43.723)	−2.657	0.008
TNF-α[M(P25–P75),pg/mL]	5.235 (4.736,9.223)	4.969 (4.398,5.851)	−1.999	0.046

Data were presented as median (25th percentile, 75th percentile); SMPP, severe *mycoplasma pneumoniae* pneumonia; MMPP, mild *mycoplasma pneumoniae* pneumonia; HMGB-1, high mobility group box 1; MCP-1, monocyte chemoattractant protein-1; TNF-α, tumor necrosis factor-α; N, neutrophils; MCP, monocyte chemoattractant protein; IL, interleukin; TF, transcription factor.

## 4 Discussion

This study prospectively included 445 children with MPP. The results showed that 255 cases (57.3%) developed SMPP, indicating that SMPP is not uncommon among hospitalized children. Early identification of high-risk children is therefore of significant clinical importance. By comparing the clinical and laboratory characteristics of mild and severe cases, we found that wheezing, shortness of breath, fever, elevated D-dimer, elevated CRP, and older age were all significantly associated with SMPP. The constructed nomogram model demonstrated good discrimination and calibration abilities, suggesting that the combined application of these clinical indicators can be used for early risk stratification of SMPP.

The present research revealed that the age of children with SMPP was markedly higher than that of those with MMPP, and the incidence of SMPP was highest in school-aged children, which is consistent with previous studies (7, 8).This finding may be attributable to the more robust immune response in older children. When MP infects these children, their immune system is triggered, producing a substantial number of inflammatory cells and cytokines, increasing the likelihood of excessive immune-inflammatory reactions and subsequent SMPP development.This study found that there was a statistically significant difference in the proportion of fever across the SMPP research samples and the MMPP research samples. In contrast, children with MMPP exhibited a higher propensity to possess moderate fever, but high fever was also relatively common. Children with SMPP exhibited a higher propensity for high fever, whereas children with MMPP more frequently presented with moderate fever. In contrast, children with MMPP exhibited a higher propensity to possess moderate fever, but high fever was also relatively common. As the peak temperature increased, the risk of developing SMPP rose accordingly. The duration and peak temperature of fever in the SMPP group were considerably longer and elevated than those in the MMPP group, which is consistent with previous studies (7). The duration of fever in children with SMPP is often longer than that in children with MMPP. This may be related to the persistence of MP infection in the body and the vigorous immunological response, which drives disease progression of SMPP. The present research revealed that the percentage of children with wheezing in the SMPP group was markedly greater than the MMPP group. During MP infection, children are more likely to have excessive airway secretion and airway hyperresponsiveness, which can trigger wheezing. The relationship between wheezing and pneumonia has been extensively documented in the literature, some studies have shown that wheezing attacks make children more prone to pneumonia (9). MPP has the potential to induce a systemic inflammatory reaction within the body, resulting in immune system dysregulation. This can provoke asthma exacerbations, intensify airway hyperresponsiveness, and in severe cases, lead to the development of severe or treatment-resistant asthma (10). This may be because after MP infects the respiratory tract, airway mucosa is damaged, causing airway obstruction and leading to wheezing. Airway mucosa damage is also one of the main factors causing airway hyperresponsiveness, which further aggravates wheezing and thus worsens the condition. Clinicians should therefore implement prompt management of MP infection to mitigate MP-induced wheezing and airway hyperresponsiveness, thereby reducing MP-related lung function impairment.

Compared with children with MMPP, CRP and D-D levels in children with SMPP elevated, and were identified as independent risk factors for SMPP. Liu et al. found that CRP > 9.74 mg/L constituted an independent risk factor for SMPP (11). Zhang et al. found that D-dimer > 0.64 μg/mL played a significant role in the assessment of SMPP (12). CRP, as an acute phase reactant, is often used as a sensitive serum biomarker to monitoring inflammatory diseases (13). Elevated CRP levels suggest that as the severity of the disease gradually increases, the body's reactivity also shows a corresponding enhancement trend. MP can directly damage the lung epithelial cell membrane, and triggering the release of pro-inflammatory cytokines such as IL-6, which overstimulates the hepatocytes to secrete CRP, causing a series of more intense inflammatory responses. The increase in D-D levels may indicate a hypercoagulable state in children with MPP, which is another typical feature of SMPP and might be associated with an overactive inflammatory cascade. MP infection causes damage to vascular endothelial cells. Damaged vascular endothelium releases various procoagulant substances and reduces the levels of natural anticoagulants in the body, disrupting the balance of the coagulation-fibrinolysis, leading to hypercoagulability, microthrombosis, and exacerbation of the progression of the disease (14).

Prior research endeavors have reported that a substantial quantity of cytokines are released in the blood of patients with SMPP and RMPP (15, 16). In our team's previous research, through the analysis of the whole blood transcriptome of SMPP and MPP children, it was found that the DEGs genes of SMPP children were mainly enriched in immune inflammation and cytokine signaling pathways. Cytokines could potentially serve as novel therapeutic targets for MPP management. Based on the previous transcriptome analysis results of our research group, this study screened out cytokines with significant differences in expression levels and measured the selected cytokines with significant differences. It was found that the levels of HMGB-1, TFEB, IL-6, -18, TNF-α, MCP-1, -2, -3, and -4 in the serum of SMPP children were significantly higher than those in the MMPP group, indicating that these inflammatory mediators are involved in the pathogenesis of SMPP, which is consistent with the study by Ding Y et al. (17). This may be due to the fact that after MP infection of the body, macrophages produce TNF-α to increase the inflammatory response, and by inducing T cell differentiation, stimulate mononuclear macrophages and lymphocytes to produce cytokines such as IL-6, increasing the concentration of IL-6 and TNF-α in the serum. A study from South Korea found that concentration of IL-18 in RMPP children was markedly elevated than that in non-RMPP. When the serum IL-18 ≥ 430 pg/mL, the sensitivity for diagnosing RMPP children was 73% and the specificity was 80%, making it a key biomarker for predicting RMPP (18). Lai et al. demonstrated in a mouse model that depletion of macrophages (rather than neutrophils) significantly reduced the host's ability to clear MP infection in the lungs, highlighting the key role of macrophages in controlling MP infection (19, 20). Lee et al. found that the levels of MCP-1 and -2 in the serum of RMPP children were markedly elevated than those in no-RMPP children, and their levels significantly decreased after treatment with azithromycin (21). This study found that MCP-1, 2, 3, and 4, as monocyte/macrophage activators, were significantly elevated in the serum of SMPP children, further confirming the involvement of macrophages in the pathogenesis and progression of SMPP. HMGB-1 is a typical damage-associated molecular pattern (DAMP) that can be released following infection or cellular injury, amplifying the inflammatory cascade. Studies have shown that after the body is infected with MP, the acquired respiratory distress syndrome toxin stimulates the release of HMGB-1. HMGB-1 activates various downstream signaling pathways through the TLR2/CD14/myeloid differentiation factor 88 (MyD88) pathway (22). Its elevation suggests more pronounced tissue damage and enhanced inflammation in children with SMPP， consistent with the Ding Y et al. (23). As a key pro-inflammatory cytokine, TNF-α promotes the recruitment of inflammatory cells, endothelial injury, and persistent pulmonary inflammation, and its increase is closely associated with severe infectious conditions (24). MCP-1, a monocyte chemoattractant protein, facilitates the migration of monocytes/macrophages into the lungs, thereby enhancing local inflammatory responses (25, 26). Elevated MCP-1 levels indicate stronger immune cell infiltration and an intensified inflammatory process in SMPP. This may be attributed to the excessive release of MCP chemokines after MP infection, which drives a large influx of monocytes/macrophages into the lung tissue; in the local hyperinflammatory environment, the protective autophagy function of the infiltrating cells is depleted, leading to cell damage and the release of endogenous danger signals such as HMGB-1 and TFEB, thereby amplifying the inflammatory cascade reaction and forming a vicious cycle.

Based on previous literature, *Mycoplasma pneumoniae* infection can activate the host immune response through TLR-related signals and induce the release of various inflammatory factors (27). Liu et al. recently further confirmed using a TLR4 gene knockout mouse model that MP infection promotes TFEB expression and its nuclear translocation via the TLR4 signaling pathway, thereby initiating protective autophagy to eliminate pathogens (28). We speculate that in the excessive inflammation environment of SMPP, the continuous activation of TLR4 can lead to the overload and eventual failure of TFEB-mediated protective autophagy, causing macrophages to undergo pyroptosis mediated by Gasdermin D (29). The intracellular TFEB is passively released into the blood. Serum TFEB may transform from a protective autophagy regulatory factor to a candidate DAMP reflecting the scale of the catastrophic death of immune cells.Although this study did not directly investigate the mechanism of TFEB, the elevated serum TFEB levels in the severe group suggest that it may serve as a systemic marker reflecting host stress, autophagy imbalance, or amplified inflammation. Combined with increased levels of HMGB-1, TFEB, IL-6, -18, TNF-α, MCP-1, -2, -3, and -4, it can be inferred that the development of SMPP is not driven by a single inflammatory factor, but rather by a complex network involving “pathogen stimulation—inflammatory chemotaxis—tissue damage—immune dysregulation.

## 5 Conclusion

This study demonstrates that wheezing, shortness of breath, fever, older age, and elevated D-dimer and CRP levels are independent risk factors for SMPP in children. A predictive nomogram incorporating these six variables was constructed, which exhibited favorable discrimination and excellent calibration, providing a quantitative tool for early risk stratification of SMPP in hospitalized children. In the cytokine sub-cohort, serum levels of HMGB-1, TFEB, IL-6, -18, TNF-α, MCP-1, -2, -3, and -4, were significantly elevated in the SMPP group; their independent predictive value requires further investigation in larger cohorts.

## 6 Limitation

This study has several limitations. First, the single-center inpatient cohort design introduces inherent selection bias, as reflected by the exceptionally high prevalence of SMPP (57.3%). This reflects the tertiary referral nature of our institution, where severe and refractory cases are concentrated. Consequently, the nomogram is calibrated for a high-risk inpatient population and will substantially overestimate SMPP risk if directly applied to general pediatric wards or outpatient settings without recalibration. Second, the cytokine sub-cohort had a limited sample size (*n* = 84, with only 42 SMPP events), which did not meet the events-per-variable (EPV) criterion for multivariable logistic regression. Therefore, cytokine-based multivariable analyses and predictive nomograms were not performed; cytokine data are presented as descriptive comparisons with univariate ROC analyses only. The identified cytokine alterations should be regarded as hypothesis-generating rather than confirmatory. Third, all serum samples were collected at a single time point within 24 h of admission; longitudinal monitoring of cytokine dynamics may provide additional predictive information. Fourth, external validation in multicenter, large-sample prospective cohorts with a more representative case mix is mandatory before clinical implementation of the nomogram.

## Data Availability

The original contributions presented in the study are included in the article/Supplementary Material, further inquiries can be directed to the corresponding author.
