# Supplementary material for: Clinical characteristics and cytokine profiles for early prediction of severe Mycoplasma pneumoniae pneumonia in children: a prospective cohort study
Source: Front Pediatr. 2026 Jul 10;14:1862535. doi: 10.3389/fped.2026.1862535 (PMC13395776; doi:10.3389/fped.2026.1862535)
Supplement: Supplementary file 1 [file Table1.docx]

Table S1 Comparison of demographic and clinical characteristics between the complete cohort (n=445) and the cytokine sub-cohort (n=84)

| Parameter | MPP（n=445） | Cytokine sub-cohort(n=84) | Statistic (T/χ2) | P value |
| --- | --- | --- | --- | --- |
| Age($\overline{\text{x}}$±s，year) | 6.64±2.62 | 5.88±3.23 | -2.637 | 0.009 |
| Stage of age[n(%)] |  |  | 15.813 | <0.001 |
| Infancy and toddler stage | 24(9.4) | 43(22.6) |  |  |
| Preschool stage | 60(23.5) | 45(23.7) |  |  |
| school stage | 171(67.1) | 102(53.7) |  |  |
| Gender |  |  | 0.295 | 0.587 |
| Male | 118（46.3） | 83（43.7） |  |  |
| Female | 137（53.7） | 107（56.3） |  |  |
| Weight ($\overline{\text{x}}$±s，kg) | 22.93±8.93 | 22.48±11.92 | -0.45 | 0.653 |
| WBC$(\overline{\text{x}}$±s,×109/L) | 8.92±4.25 | 9.18±3.94 | -0.532 | 0.595 |
| N($\overline{\text{x}}$±s,%) | 60.73±14.87 | 58.46±13.77 | 1.299 | 0.194 |
| CRP($\overline{\text{x}}$±s,mg/L) | 1.10±1.92 | 0.99±1.27 | 0.530 | 0.597 |
| D-D(($\overline{\text{x}}$±s,,ug/ml) | 21.64±27.47 | 19.29±19.66 | 0.747 | 0.455 |
| PCT(($\overline{\text{x}}$±s,ng/ml) | 0.24±0.57 | 0.27±0.92 | -0.411 | 0.681 |
| fever duration （$\overline{\text{x}}$±s，day） | 4.30±3.07 | 4.13±3.12 | 0.468 | 0.640 |
| peak temperature($\overline{\text{x}}$±s，℃) | 2.12±1.08 | 1.96±1.01 | 1.203 | 0.229 |

Data are presented as the mean ± standard deviation, or the number (percentage). MMPP: *mycoplasma pneumoniae* pneumonia;PCT:Procalcitonin,WBC:white blood cell, D-D:D-dimer,N:Neutrophils,CRP:C-reactive protein，N：neutrophilic granulocyte percentage
